# Incorporation of Phosphorus Impurities in a Silicon Nanowire Transistor with a Diameter of 5 nm

**DOI:** 10.3390/mi10020127

**Published:** 2019-02-15

**Authors:** Yanfeng Jiang, Wenjie Wang, Zirui Wang, Jian-Ping Wang

**Affiliations:** 1 Department of Electrical Engineering, IoT College, Jiangnan University, Wuxi 214122, China; wdz@ncut.edu.cn (W.W.); hightoppub@126.com (Z.W.); 2 Department of Electrical & Computer Engineering, University of Minnesota, Minneapolis, MN 55414, USA; jiangy@umn.edu

**Keywords:** doping incorporation, plasma-aided molecular beam epitaxy (MBE), segregation, silicon nanowire

## Abstract

Silicon nanowire (SiNW) is always accompanied by severe impurity segregation and inhomogeneous distribution, which deteriorates the SiNWs electrical characteristics. In this paper, a method for phosphorus doping incorporation in SiNW was proposed using plasma. It showed that this method had a positive effect on the doping concentration of the wires with a diameter ranging from 5 nm to 20 nm. Moreover, an SiNW transistor was assembled based on the nanowire with a 5 nm diameter. The device’s I_ON_/I_OFF_ ratio reached 10^4^. The proposed incorporation method could be helpful to improve the effect of the dopants in the silicon nanowire at a nanometer scale.

## 1. Introduction

Semiconductor nanowire (NW) shows potential for its application as a fundamental building block for nano-electronic and nanophotonic devices. It also offers substantial promise for integrated nanosystems [1,2,3]. The transistors based on the nanowires are attracting increasing attention due to their potential applications in electronics and biomolecule detection [4]. Until now, many works on the transistors assembled using semiconductor nanowires [3,4,5,6] have been reported. Promising device characteristics, such as high hole/electron mobilities, large ON-currents, large I_ON_/I_OFF_ ratio, and good subthreshold swings, are shown on the fabricated devices [1,2,3].

Among the proposed various nanowires, silicon nanowire (SiNW) is considered as the most promising candidate as it shows excellent characterizations, and it is compatible with the main-stream technology of the integrated circuit. For a transistor based on silicon nanowire to be used in a modern integrated circuit, its size should be scaled down continuously while keeping its electrical characteristics. In this way, the silicon nanowire transistor could be a strong candidate as the building block for an advanced integrated circuit to follow the Moore’s Law in the Post-Moore Era [3,7]. A silicon nanowire transistor with a smaller diameter can have a higher density, smaller capacitance, more transconductance, and controllability of the gate voltage on drain current. Thus, it is meaningful to explore the SiNW transistor with a smaller diameter.

However, there are some unsolved problems encountered during the miniaturization process. One of the key issues is the doping incorporation in the nanowire. Based on the experimental results in Reference [8], the conductivity of the doped SiNW with diameter of less than 20 nm is much lower than the predicted conductivity based on the actual doping concentration. This means part of the impurities in the wire are not incorporated when the diameter of the SiNW is smaller than 20 nm. Some theories were proposed to explain the discrepancy [9,10,11,12], where the basic ideas included the influences of the doping profiles [9], surface states [10,11], and diameter variation [12] of the thin wire. For example, it was demonstrated that the surface state of the nanowire was very sensitive to the variation of the diameter along the length direction of the nanowire. Thus, the surface state was influenced by the diameter. Then, the carrier concentration and carrier’s lifetime were changed accordingly [11]. Mikael et al. [13] reported the direct observation of the influence of the segregation of the impurities in the silicon nanowire. An abrupt decrease of the free charge carrier concentration was observed on the nanowire with a diameter smaller than 15 nm. Therefore, the deterioration in the effective carrier concentration of the nanowire with a diameter in tens nanometers was a severe problem, which could influence the characteristics of the SiNW transistor at a nanometer scale.

Until now, only a few works on how to incorporate the impurity atoms in silicon nanowires were reported. Some researchers used the annealing method to incorporate the impurities into the nanowires by tuning the interface states [14,15]. In this paper, a plasma-assisted molecular beam epitaxial (MBE) method was proposed for the preparation of the nanowires with a diameter varied from 5 nm to 20 nm. It was demonstrated that the plasma could help to increase the conductivities of the doped nanowires. The SiNW transistor with 5 nm diameter was fabricated based on the prepared nanowire and it showed good characteristic results.

## 2. Materials and Methods

The growth system was a molecular beam epitaxy (MBE) apparatus (Riber 32), with a base pressure below 5 × 10^−11^ Torr, including electron-beam guns for the evaporation of Au and Si, as well as a substrate heater. A radio frequency plasma source was equipped to assist the growth of the nanowires. The growth substrate was boron-doped Si (111) wafer. It was cleaned in a hot acetone bath for 10 min and subsequently etched in a NH_4_OH: H_2_O_2_:H_2_O (1:1:5) solution for 10 min at 70 °C, and then dipped in a HF:H_2_O (1:5) solution for 10 s. After the cleaning process, the wafer was loaded onto a sputtering system. Silicon wafer was ion bombarded in the system. The substrate was irradiated with 120 keV Ar ions. Energy of 120 keV was chosen because the projected range of the ions with such energy created a 2-nm roughness on the surface. The generated roughness was helpful for the formation of the Au droplets. The sizes of the Au droplets would determine the diameters of the grown silicon nanowires afterwards. The periodic rough surface can lead to a good templating of Au droplets.

After the sputtering, the substrate was loaded onto the MBE system. The silicon substrate was annealed in an ultra-high vacuum at 925 °C for 15 min, to obtain an oxygen-free surface. The 7 × 7 reconstruction was obtained after annealing of the substrate, as checked by low-energy electron diffraction (LEED). The temperature of the substrate was controlled by a thermocouple, as well as a pyrometer.

Au film was deposited at a substrate at room temperature, with a thicknesses between 1 nm and 1.2 nm as measured by a quartz monitor. After that, the temperature was increased to 525 °C. The technique utilizing spontaneous dewetting can engineer patterns in soft materials on the nanometer scale, without the conventional lithographic process [14]. The sizes of the formed Au droplets were not uniform because of the existence of the roughness of the silicon substrate. Given the diameters of the grown nanowires depended on the sizes of the Si–Au alloy droplets, the silicon nanowires with different diameters were grown simultaneously on the same substrate.

During the SiNW growth, constant Si flux amounted to 4.7 Å/s. A solid phosphorus source with 99.9995% purity was used, where the concentration was related to the source’s temperature. Based on the calibrated method by Pratyush, et al. [15], the source temperatures were monitored between 900–1200 °C to obtain concentrations varying from 1 × 10^16^ cm^−3^ to 1 × 10^19^ cm^−3^. The evaporation time lasted for 1.8 h.

For the convenience of comparison, two groups of samples with diameters from 5 nm to 20 nm were prepared. Group A was grown with plasma-assistance while group B was without plasma. Except the plasma, the other parameters during the growth of the two sets of the SiNWs were all the same. The plasma source was 250 W radio frequency (RF) power, with a frequency of 13.56 MHz. In this way, the two groups of samples were prepared using the same conditions, except that group A was with plasma assistance while the group B was without plasma. After the substrates were loaded out, the gold caps on the top of the grown SiNWs were removed using an aqueous solution of KI and I_2_.

## 3. Results

**Figure 1:**
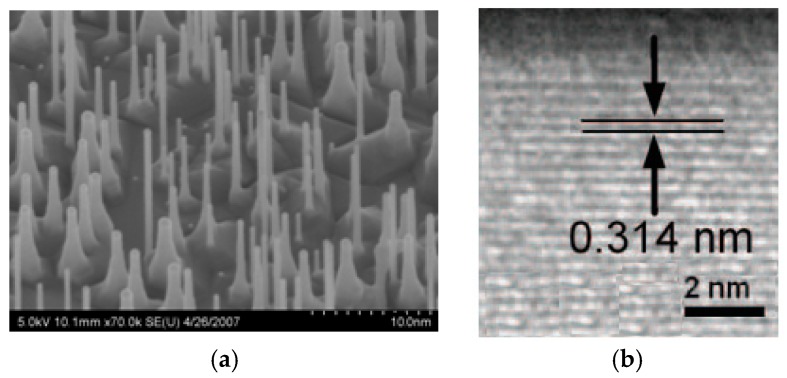
(**a**) Scanning electron microscopy (SEM) photograph of the grown silicon nanowires (SiNWs). Diameters are varied from 5 nm to 20 nm. The scale bar is 100 nm. (**b**) Transmission electron microscopy (TEM) photograph of the silicon nanowire, showing its crystal structure in the <111> direction. The scale bar is 2 nm.

The scanning electron microscopy (SEM) image and high resolution transmission electron microscopy (HRTEM) are shown in Figure 1. A bunch of the grown SiNWs can be seen clearly, with diameters from 5 nm to 20 nm, as shown in Figure 1a. The TEM image in Figure 1b shows the grown nanowire with a single-crystalline structure and vertically along the <111> crystalline direction.

**Figure 2:**
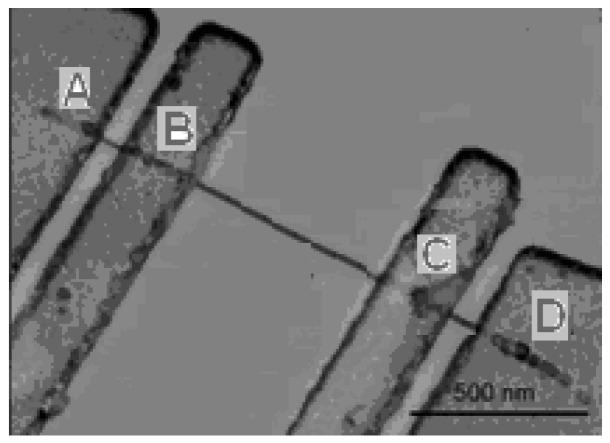
Image of the electrical contacts of the prepared SiNW. Scale bar is 500 nm.

The prepared SiNWs were put into ethanol for ultrasonic dispersion. Afterwards, the wires were transferred onto the SiO_2_/Si substrate. Multiple electrical contacts as shown in Figure 2 were used for characterization of the prepared wires.

**Figure 3:**
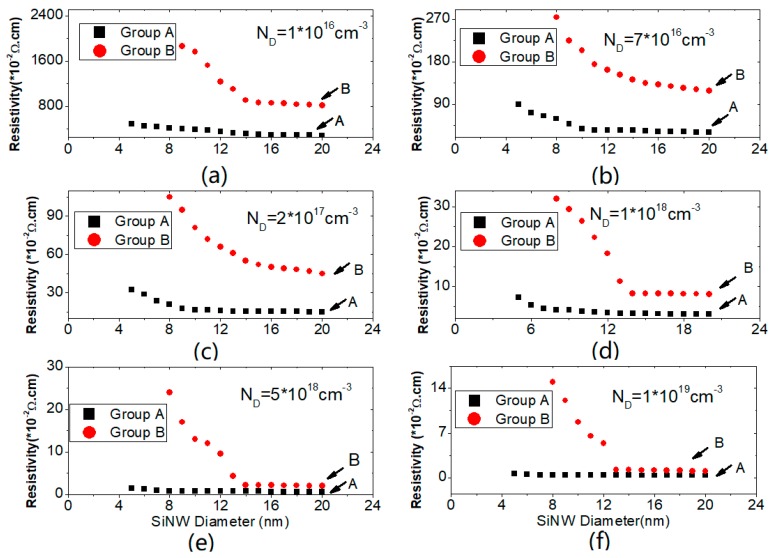
The measured resistivities of the SiNWs with different diameters for groups A and B. (**a**) N_D_ = 1 × 10^16^ cm^−3^; (**b**) N_D_ = 7 × 10^16^ cm^−3^; (**c**) N_D_ = 2 × 10^17^ cm^−3^; (**d**) N_D_ = 1 × 10^18^ cm^−3^; (**e**) N_D_ = 5 × 10^18^ cm^−3^; (**f**) N_D_ = 1 × 10^19^ cm^−3^.

Characterizations were conducted to obtain the resistivities with diameters from 5 nm to 20 nm for both groups (A and B), corresponding to the different concentrations, as shown in Figure 3. It could be clearly observed that the resistivities of the SiNWs in group A, with plasma assistance during growth, were much lower than those in the group B without plasma assistance. For group B, the resistivities remained at almost the same level from 20 nm to 13 nm with different doping concentrations. However, for the wires smaller than 12 nm, the resistivities increased dramatically, corresponding to the decrease of the number of the incorporated carriers. When the diameters were smaller than 8 nm, the resistivities of group B were unreadable, as is not shown in Figure 3. The results coincide with other reported results [13,16,17,18]. Theoretical explanations on this phenomenon could be attributed to quantum confinement [16], surface segregation of dopants [17], or ionization energy due to a dielectric mismatch at the wire surface [18]. With the diameter decreasing, there will be an increased possibility of the occurrence of surface depletion of charge carriers due to interface states, trapped charges, changeable mobilities, and a size-dependent incorporation of dopants during growth. Therefore, the amount of the incorporated dopants in the SiNW could be decreased [13].

Compared with the results of group B, group A showed much better results, as shown in Figure 3. For all the six different concentrations ranging from 1 × 10^16^ cm^−3^ to 1 × 10^19^ cm^−3^, the resistivities always remained the same with the diameters changing from 20 nm to 10 nm. Slight increments occured on the samples with diameters from 10 nm to 5 nm. This demonstrated that the segregation effect was alleviated by using the plasma during growth. It was directly observed that the plasma assistance could be helpful in incorporating the impurities in the SiNWs with diameters down to 5 nm.

The fabricated 5-nm SiNW was used to assemble the transistor. The source and drain electrodes were defined by the lithography process. The grown SiNWs were transferred onto the electrodes using Cui’s approach [1]. The metal contacts were evaporated on the electrodes. The naturally grown oxide layer on the SiNWs can be used as the gate oxide.

**Figure 4:**
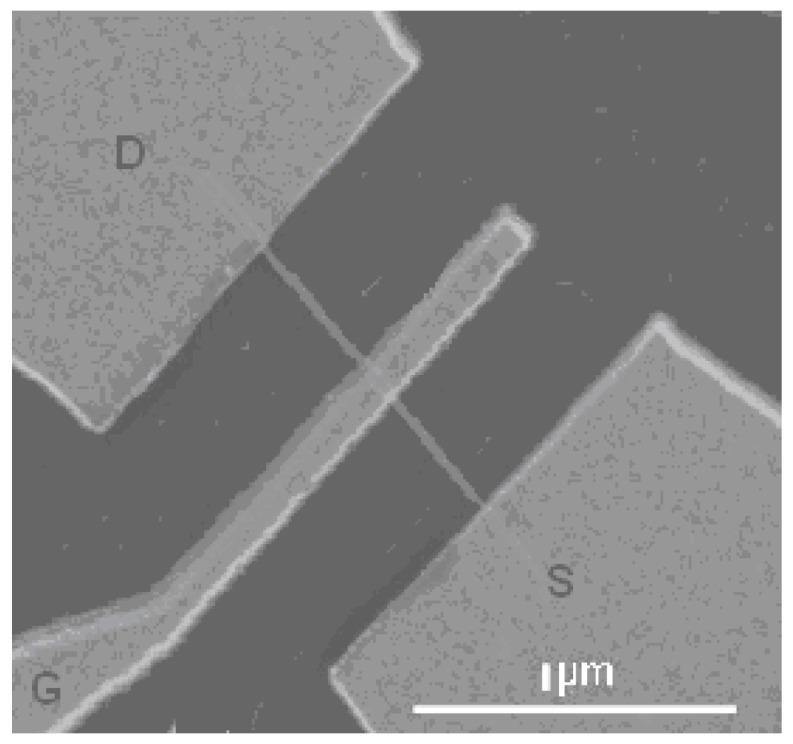
SEM image of the 5 nm SiNW transistor. Scale bar is 1 μm.

Figure 4 shows the SEM photograph of the transistor, where the drain, source, and gate are annotated. N.Singh et al. [7] showed the transistor with a diameter ≤5 nm using the top-down method. In this study, the 5-nm SiNW transistor was fabricated using the “bottom-up” approach. The following characterizations on the SiNW transistor were based on the prepared 5-nm-wire with 2 × 10^17^ cm^−3^ doping concentration.

**Figure 5:**
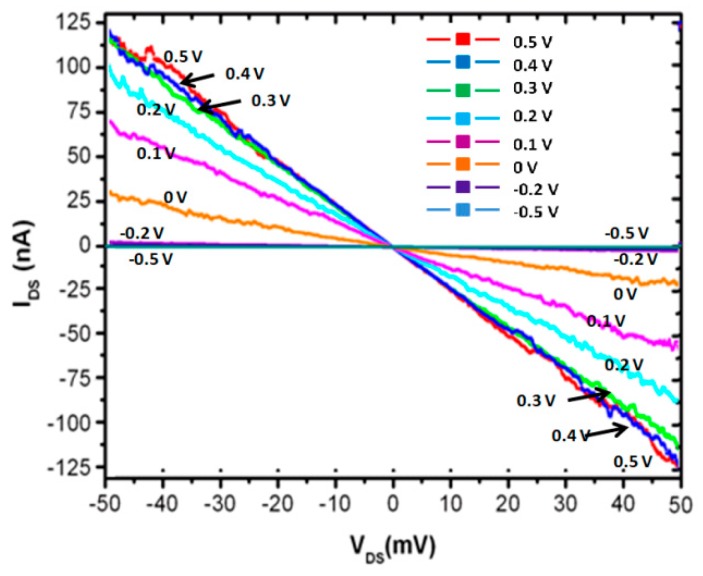
I_DS_-V_DS_ relationships of the 5 nm SiNW transistor with different V_G_ voltages, ranging from −0.5 V to 0.5 V.

Figure 5 shows the electrical properties of the I_DS_-V_DS_ with different gate voltages ranging from −0.5 V to 0.5 V. It shows a linear response characteristic of the I_DS_-V_DS_, indicating ohmic terminal contacts. When V_G_ = 0 V, the I_DS_-V_DS_ curve is linear with a resistance of 2 MΩ. When V_G_ > 0 V, the I_DS_-V_DS_ curves still remain linear, whereas the resistance decreases with the gate voltage rises. When V_G_ = 0.5 V, the resistance is 0.42 MΩ. Nonlinear relationships of the I_DS_-V_DS_ exhibit at V_G_ < 0. Thus, it can be seen that the controllability of V_G_ on the transistor is sensitive.

**Figure 6:**
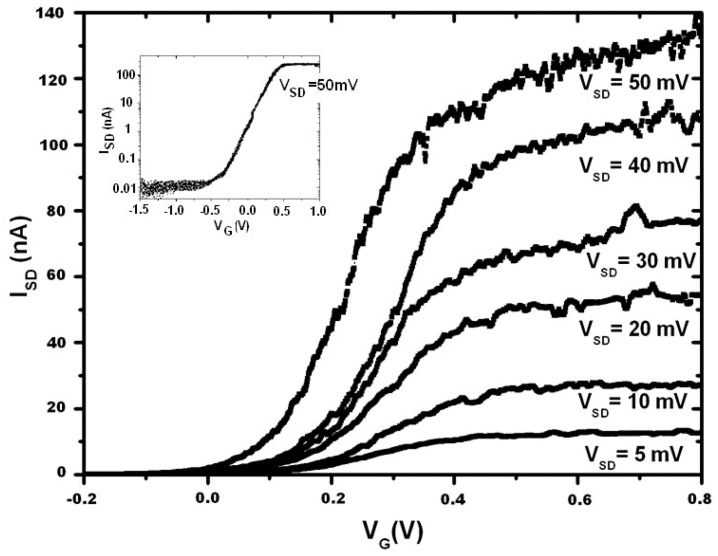
I_SD_-V_G_ relationship of the 5-nm SiNW transistor, with V_SD_ sweeping from 5 mV to 50 mV. Inset shows the algorithm relationship of I_SD_-V_G_ when V_SD_ = 50 mV, where the I_ON_/I_OFF_ ratio is 10^4^.

Figure 6 shows the relationship between the drain current versus the gate voltage (I_SD_-V_G_), with V_SD_ ranging from 5 mV to 50 mV. The inset shows the I_SD_-V_G_ characteristic in an algorithm scale with V_SD_ = 50 mV. It can be clearly seen that the I_ON_/I_OFF_ ratio reaches 10^4^.

## 4. Discussion

The growing process is based on the ion bombardment on the silicon substrate. Roughness is generated on the surface. Thus, the tension remains on the deposited Au layer. A post-annealing step can help to generate nanosized Au-Si droplets. The following growth of the SiNWs is directly influenced by the droplets. The diameters of the grown SiNWs are determined by the dimensions of the droplets. As such precise control on the size of the droplets is critical for the grown SiNWs. Figure 1a shows that the diameters of the grown NWs are randomly varied from 5 nm to 20 nm. This is helpful for the experiment in this paper, since a several samples with different diameters can be prepared on the same substrate at the same time, with the same conditions. For future application in integrated circuits, it is important to uniformly prepare the SiNWs. To satisfy the mass production requirement, an advanced ultrafine lithographic process can be adopted instead of the ion bombardment approach.

**Figure 7:**
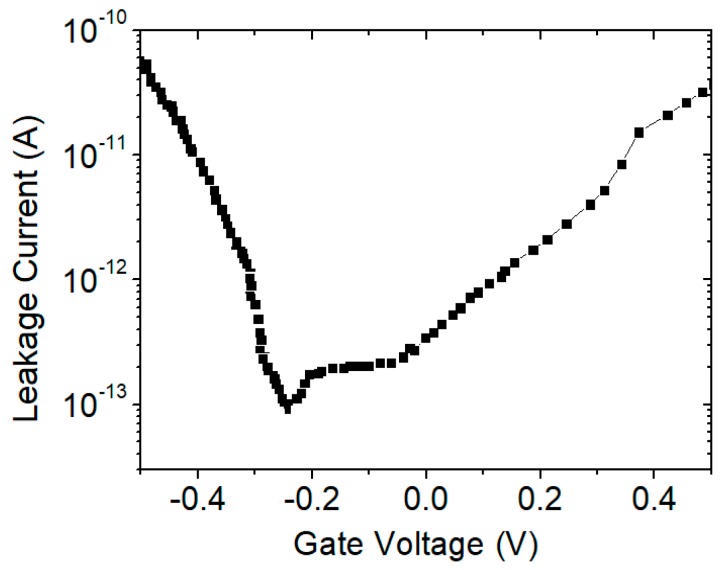
Leakage current versus gate voltage for the prepared SiNW transistor. Scanning was performed from −0.5 V to 0.5 V.

For the fabricated SiNW transistor, its gate oxide layer was grown naturally after the SiNW was extracted from the MBE system. During the subsequent annealing and electrode deposition steps, the oxide layer was gradually accumulated on the surface. To verify the quality of the oxide layer, the leakage current was measured on the prepared 5-nm SiNW transistor. The result is shown in Figure 7, showing the characteristics of the leakage current versus the gate voltage. Scanning was performed from −0.5 V to 0.5 V. It can be seen that the leakage current was less than 0.1 nA, demonstrating the quality of the oxide layer.

**Figure 8:**
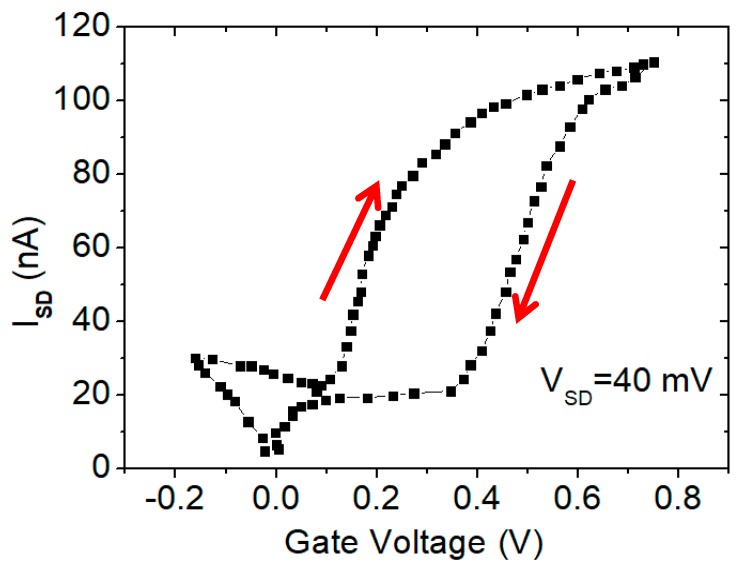
Transfer characteristic of the 5-nm SiNW transistor, showing a hysteresis value of ~0.2 V at a 40 mV drain voltage.

To investigate the states of the trapped charges in the fabricated 5-nm SiNW transistor, the hysteresis behavior was characterized on the device. Hysteresis is the shift in the threshold voltage during the forward (+V to −V) and the reverse gate voltage sweep (−V to +V), at a constant drain voltage bias. The trapping is typically manifested as hysteresis behavior during transistor IV scans. Figure 8 shows the measured hysteresis loop of the transistor at the drain voltage V_SD_ = 40 mV. The threshold voltages in the down-sweep stage were higher than the voltages in the up-sweep stage, suggesting the negative polarity of the trapped charges in the device. The trapping state scatters the charge carriers, and hence it decreases the carrier mobility. Further research work on the trapped charges is necessary to investigate the source and the locations in the device.

The current of the assembled 5-nm SiNW transistor are at a nA scale, as shown in Figure 5 and Figure 6. There are obvious noises on the IV curves in Figure 6. The origin of the noises is currently unclear. There were two possible sources of the noises, which included the experimental set-up and the fabricated device. Further investigation should be conducted to clarify the origin and to improve the quality of the transistor.

**Figure 9:**
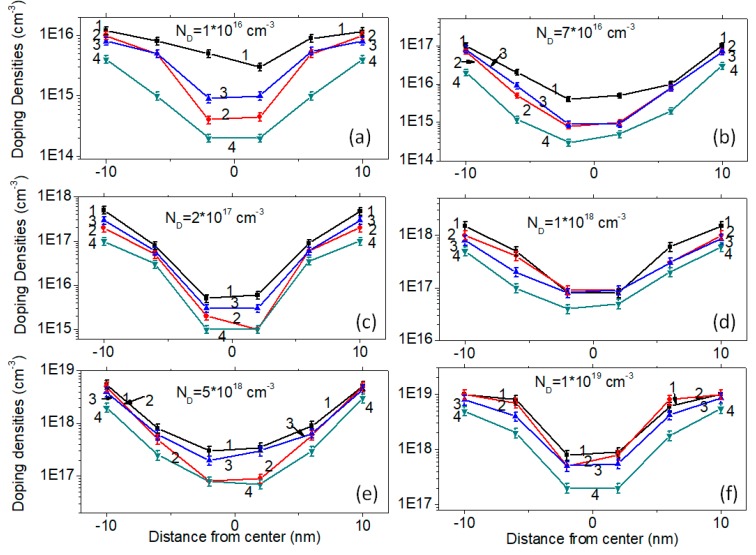
Doping densities of the grown SiNWs with diameter 20 nm. Line “1” is the chemical doping densities with plasma assistance. Line “2” is the chemical doping densities without plasma assistance. Line “3” is the electrical doping densities with plasma assistance. Line “4” is the electrical doping densities without plasma assistance. (**a**) N_D_ = 1 × 10^16^ cm^−3^; (**b**) N_D_ = 7 × 10^16^ cm^−3^; (**c**) N_D_ = 2 × 10^17^ cm^−3^; (**d**) N_D_ = 1 × 10^18^ cm^−3^; (**e**) N_D_ = 5 × 10^18^ cm^−3^; (**f**) N_D_ = 1 × 10^19^ cm^−3^.

To investigate the doping densities of the grown nanowires, the capacitance-voltage technique [12] was used to measure the doping profiles of the samples, with and without plasma assistance. The 20-nm SiNWs were used for the characterization. The results are shown in Figure 9, using the six different doping densities. Four results were summarized as corresponding to one single doping level, which included the chemical doping densities with plasma assistance (Line 1), the chemical doping densities without plasma assistance (Line 2), the electrical doping densities with plasma assistance (Line 3), and the electrical doping densities without plasma assistance (Line 4). It could be seen that there were obvious discrepancies among the four lines for one single doping level. As shown in Figure 9a, the doping level was N_D_ = 1 × 10^16^ cm^−3^. For the nanowire prepared using plasma assistance (Line 1), its chemical doping concentration on the surface was 1.2 × 10^16^ cm^−3^. It drops at the center, to around 5 × 10^15^ cm^−3^. For the nanowire prepared without plasma assistance (Line 2), its chemical doping concentration on the surface was 1 × 10^16^ cm^−3^. Its center density was 5 × 10^14^ cm^−3^. Comparing the data in Line 1 and Line 2, it could be seen that the plasma assistance was helpful for the doping distribution, assisting the distribution more homogeneously. For the five other doping concentrations, the same tendency could be found in which the chemical doping profiles assisted by plasma showed more homogeneity than those without plasma assistance.

The actual electrical doping densities for the sample with N_D_ = 1 × 10^16^ cm^−3^ are shown in Figure 9a. Line 3 corresponds to the electrical doping profile with plasma. It showed the same tendency as that of the chemical doping profile (Line 1). The surface concentration was higher than that at the center. At the same position, the electrical density was smaller than the chemical density, demonstrating that partial dopants were not involved in the electrical transportation. However, it showed that the electrical doping concentrations with the plasma (Line 3) were obviously higher than concentrations without plasma (Line 4). This means that the plasma was helpful in improving the electrical doping concentrations in the grown SiNWs. The same conclusion could be made for the other samples with different chemical concentrations, as shown in Figure 9b–f.

For the silicon nanowire, p- or n-type nanowires can be manufactured when suitable impurities are doped in it. However, the question of how the electrical conductivity depends on the doping level remains largely open. A prominent part of the doping atoms has been blocked because of the existence of surface states, trapped charges, or defects, et al. [9,11]. The segregation of the doping atoms appears more obvious when the diameter gets smaller. This means that the modulation of the doping concentration on the nanowire’s conductivity is not as sensitive as anticipated. For the potential application of SiNWs with a smaller diameter, the efficiency of the doping concentration on the modulation of conductivity should be increased.

Most of the related works have used high impurity concentrations, even near or above the Mott density corresponding to the metal–nonmetal transition in the bulk semiconductor [18]. However, a high concentration could induce surface segregation. This may be one of the main reasons for the low conductivity. In this paper, six different concentrations ranging from 1 × 10^16^ cm^−3^ to 1 × 10^19^ cm^−3^ were considered, in which the wide range of concentrations can help us to not only observe the incorporation effect more clearly, but the surface segregation as well. The merits of this approach have been verified because the conductivities of the nanowires can be measured at a medium level with a smaller diameter.

The mechanism of the plasma assisted incorporation in this paper is still under investigation. The measured doping profiles in Figure 9 show that the surface concentrations of the samples with plasma assistance were higher than those without plasma assistance, indicating that the side wall conductance was enhanced by the plasma. I. Amit et al. [19,20] and E. Koren et al. [21] found that the side wall conductance significantly contributed to the conductivity of the nanowires. The results in this paper, as shown in Figure 9, reveal one important contribution of the plasma on the doping profiles. That is, the surface profiles with plasma are improved in terms of the doping concentrations. The side wall conductance of the SiNWs with plasma assistance, either the chemical concentrations or the electrical concentrations, are both improved.

From the above results, two interesting conclusions can be reached. Firstly, a large part of the doping impurities are incorporated into the wires using the plasma assisting method. A relative number of the segregation atoms still exist because the calculated electron mobility was smaller than that in the bulk material. However, the comparison between groups A and B showed obvious improvements. Secondly, all the nanowires in group A had measurable conductivities, even down to a 5 nm diameter. Therefore, the plasma assisting approach is effective in solving the impurity segregation issue in small nanowires down to 5 nm [14]. In this paper, the transistor assembled with 5 nm SiNW using a bottom-up approach was demonstrated as showing good transistor characteristics. Another 5 nm transistor based on the SiNW was reported by N. Singh et al. [7].

## 5. Conclusions

The method of growing SiNWs using plasma was reported in this paper. Different concentrations ranging from 1 × 10^16^ cm^−3^ to 1 × 10^19^ cm^−3^ of the SiNWs with diameters from 5 nm to 20 nm were fabricated and characterized. Compared with the SiNWs without plasma assistance, obvious improvements in the conductivities could be observed in the SiNWs with plasma assistance. By preparing the SiNW transistor based on the 5 nm nanowire, it showed good transistor characterizations. The device’s I_ON_/I_OFF_ ratio can be as high as 10^4^. This work is helpful for future work on SiNW transistors with diameters in several nanometers.
